# Clinicopathological and prognostic significance of SMAD4 in non-small cell lung cancer: A meta-analysis and database validation

**DOI:** 10.1097/MD.0000000000034312

**Published:** 2023-07-21

**Authors:** Zhiqiang Li, Yunfei Huang, Rongsheng Zhou, Zhicheng Li, Qitao Yan

**Affiliations:** a Department II of Thoracic Surgery, The Fifth Hospital of Dalian, Dalian, China.

**Keywords:** clinicopathological parameters, meta-analysis, non-small cell lung cancer, prognosis, SMAD4

## Abstract

The relationship between SMAD family member 4 (SMAD4) and the clinicopathological and prognostic significance of non-small cell lung cancer (NSCLC) patients is unclear. Our aim was to investigate the association between SMAD4 expression and clinicopathological parameters and NSCLC prognosis.

**Methods:**

We searched articles in databases from inception to July 2022 to retrieve literature related to SMAD4 expression and the clinicopathological and/or prognostic significance of NSCLC patients. Odds ratios (ORs), hazard ratios (HRs) and 95% confidence intervals (CIs) were calculated. We evaluated the expression of SMAD4 and overall survival (OS) in NSCLC using the Kaplan–Meier plotter database.

**Results:**

Eight articles with 1461 NSCLC patients were included. SMAD4 expression was related to tumor differentiation (OR = 0.359, 95% CI: 0.238–0.543, *P* = .000), lymph node metastasis (OR = 0.469, 95% CI: 0.04–0.725, *P* = .001), tumor node metastasis stage (OR = 0.238, 95% CI: 0.156–0.362, *P* = .000) and good OS (HR = 0.592, 95% CI: 0.332–0.853, *P* = .000) in NSCLC. There was no significant association between SMAD4 expression and age (OR = 0.822, 95% CI: 0.515–1.312, *P* = .411) or sex (OR = 1.056, 95% CI: 0.675–1.653, *P* = .811). Furthermore, SMAD4 expression was lower in NSCLC, and a good prognosis in NSCLC (HR = 0.6, 95% CI = 0.51–0.72, *P* = 4.2 e-9) was shown to correlate with higher SMAD4 expression using the Kaplan–Meier Plotter database.

**Conclusion:**

SMAD4 expression is lower in NSCLC and correlated with lymph node metastasis, tumor differentiation, tumor node metastasis stage and good OS for NSCLC patients.

## 1. Introduction

As one of the most frequently diagnosed malignant neoplasms, lung cancer may present a serious public health problem and societal burden.^[1]^ In 2020, there were approximately 2.3 million new cases and 1.8 million deaths of lung cancer worldwide. Although much progress in the treatment of lung cancer has been made, most lung cancer patients are already at an advanced stage when diagnosed, and the mortality rate of lung cancer patients is still high.^[2,3]^ Non-small cell lung cancer (NSCLC) is the main pathological form of lung cancer.^[4]^ Approximately 48% of NSCLC patients with advanced-stage disease have metastasis when they are initially diagnosed.^[5]^ Therefore, it is very important to detect diagnostic and prognostic markers for NSCLC.

SMAD family member 4 (SMAD4) is one of the mediators of complexes with receptor-activated SMADs. SMAD complexes regulate the expression of target genes and are involved in many processes, such as inflammation and apoptosis.^[6,7]^ Accumulative studies have shown that SMAD4 is involved in tumor invasion, metastasis and prognosis various tumors, such as cholangiocarcinoma and pancreatic cancer.^[8,9]^ Moreover, many studies have detected the clinicopathological and prognostic significance of SMAD4 expression in NSCLC patients, but the potential clinicopathological value of Smad4 in NSCLC is inconsistent. Guo et al found that SMAD4 had a strong correlation with the differentiation and lymph node metastasis of NSCLC patients.^[10]^ However, Xie et al^[11]^ found that SMAD4 expression was not related to the differentiation and lymph node metastasis of NSCLC patients. In addition, inconsistent results concerning the prognostic value of Smad4 in NSCLC were also found in different articles. Ziemke et al^[12]^ reported that SMAD4 expression was not associated with prognosis of NSCLC. Tong et al^[13]^ reported that the expression of Smad4 waw significantly correlated with prognosis of NSCLC. Therefore, we performed this study to explore the relationship between SMAD4 expression and clinicopathological parameters and prognosis of NSCLC patients.

## 2. Methods

Our study was conducted according to the guidelines of Preferred Reporting Items for Systematic Reviews and Meta-Analyses, and our study has been registered in the PROSPERO database (CRD42023409645). Our study does not involve human and animal experiments, and the Ethical approval and consent to participate was not necessary.

### 2.1. Search strategies

We searched the articles in PubMed, Elsevier, EMBASE, CNKI, WanFang, VIP, Web of Science, the Cochrane Library and the Cochrane Library with the following search terms: “cancer” (or “carcinoma”) AND “non-small cell lung cancer” (or “NSCLC”) AND “SMAD family member 4” (or SMAD4 or “DPC4” or “deleted in pancreatic carcinoma locus 4.”)

### 2.2. Study selection

We used the following inclusion criteria: articles published until July 2022; SMAD4 expression in NSCLC tissue was tested by immunohistochemistry; cases of NSCLC confirmed by pathology; and studies mentioning SMAD4 expression and clinicopathological parameters and/or prognosis of NSCLC and hazard ratios (HRs) with 95% confidence intervals (CIs) were included.

The exclusion criteria were as follows: no clinical data; repeated data, case report, letter and review; or preoperative radiotherapy and chemotherapy.

### 2.3. Data extraction and quality assessment

Z.L. and Y.H. screened the articles in the databases and analyzed the relevant articles independently. Any disagreement between the 2 authors was discussed with other authors (R.Z. and Z.L.). The PRISMA statement guidelines for conducting and reporting systematic reviews were used to select and analyze the included articles. The Newcastle–Ottawa Scale score was used to assess the quality of the included articles.

### 2.4. Kaplan–Meier Plotter database

Kaplan–Meier plotter (http://kmplot.com/analysis/) is a database that can be used to assess the effect of different genes on the prognosis of several kinds of cancer patients. SMAD4 expression in NSCLC and the relationship between SMAD4 expression and survival in NSCLC patients were analyzed. The HR and 95% CI and log-rank *P* value were calculated.

### 2.5. Statistical analysis

OR, HR, and 95% CI were used to evaluate the association between SMAD4 expression and clinicopathological parameters and prognosis of NSCLC patients with STATA 10.0 software. The evaluation of heterogeneity was performed using the Higgins *I*^2^ statistic and Cochran’s *Q* test. When *P* was < .10 and/or *I*^2^ was > 50%, the heterogeneity was considered significant, and the random-effects model was used for analysis. When *P* ≥ .10 and/or *I*^2^ < 50%, the fixed-effects model was used for analysis. Publication bias was analyzed by Begg’s test and Egger’s test, and *P* < .05 was defined as significant.

## 3. Results

### 3.1. Characteristics of the included studies

**Figure 1.**
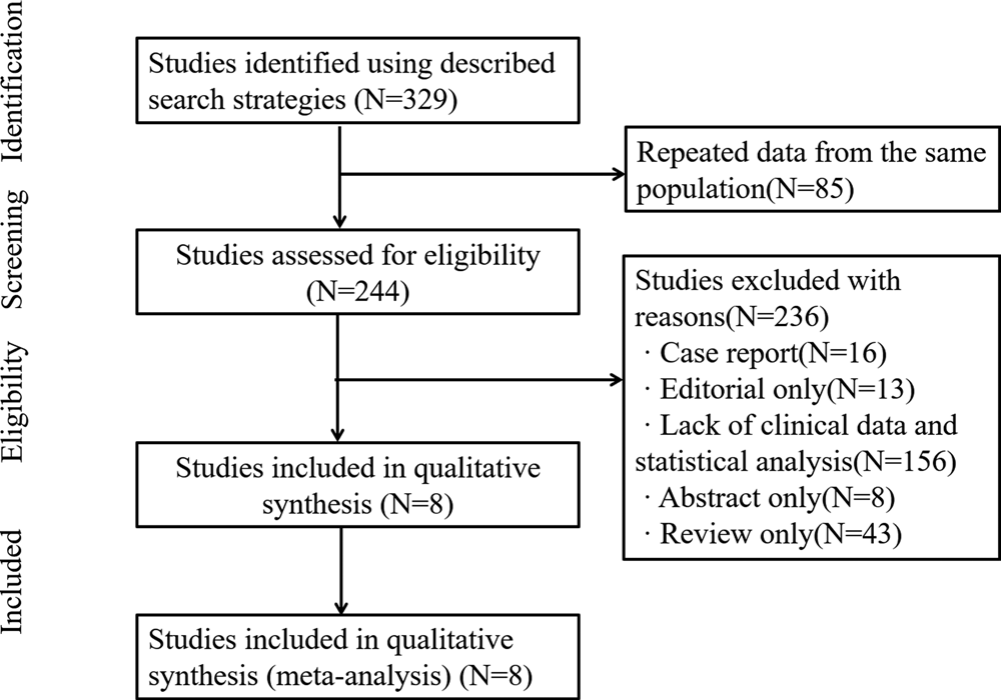
Flow diagram of the literature review.

Figure 1 shows the flow diagram of our included studies. The electronic data search yielded 329 studies. Eighty-five articles were removed because they were repeated articles. After screening the titles and abstracts, 236 articles were removed, including case reports (N = 16), editorials (N = 13), lack of clinical data and statistical analysis (N = 156), abstracts (N = 8), and reviews (N = 43). Finally, 8 studies involving 1461 NSCLC patients were included.^[10–12,14–18]^ The 8 studies were published from 2011 to 2021 and were all retrospective studies, and the sample sizes of the included studies were between 28 and 963. The information of the 8 included studies is listed in Table 1.

**Table 1:** Main characteristics and results of the eligible studies.

No.	First author	Year	No.	Magazine	During	Gender (M/F)	RCT (Y/N)	Country	Method	NOS
1	Guo	2021	52	BMC Pulmonary Medicine	Not report	31/21	N	China	IHC	9
2	Lv	2012	150	J Chongqing Medical University	2002–2004	84/66	N	China	IHC	7
3	Wang	2011	60	J Wannan Medical college	2008–2009	43/17	N	China	IHC	6
4	Wang	2020	963	LAB INVEST	20017–2019	413/550	N	China	IHC	8
5	Song	2014	71	Chin J Lab Diagn	2011–2012	53/18	N	China	IHC	6
6	Xie	2015	85	Chin J Lung Cancer	2003–2013	61/24	N	China	IHC	6
7	Ziemke	2017	28	Lung Cancer	2004–204	13/15	N	USA	IHC	7
8	Ke	2008	52	Human Pathology	2000–2003	38/14	N	China	IHC	8

NOS = Newcastle–Ottawa scale.

### 3.2. Association between SMAD4 and clinicopathological parameters

**Figure 2.**
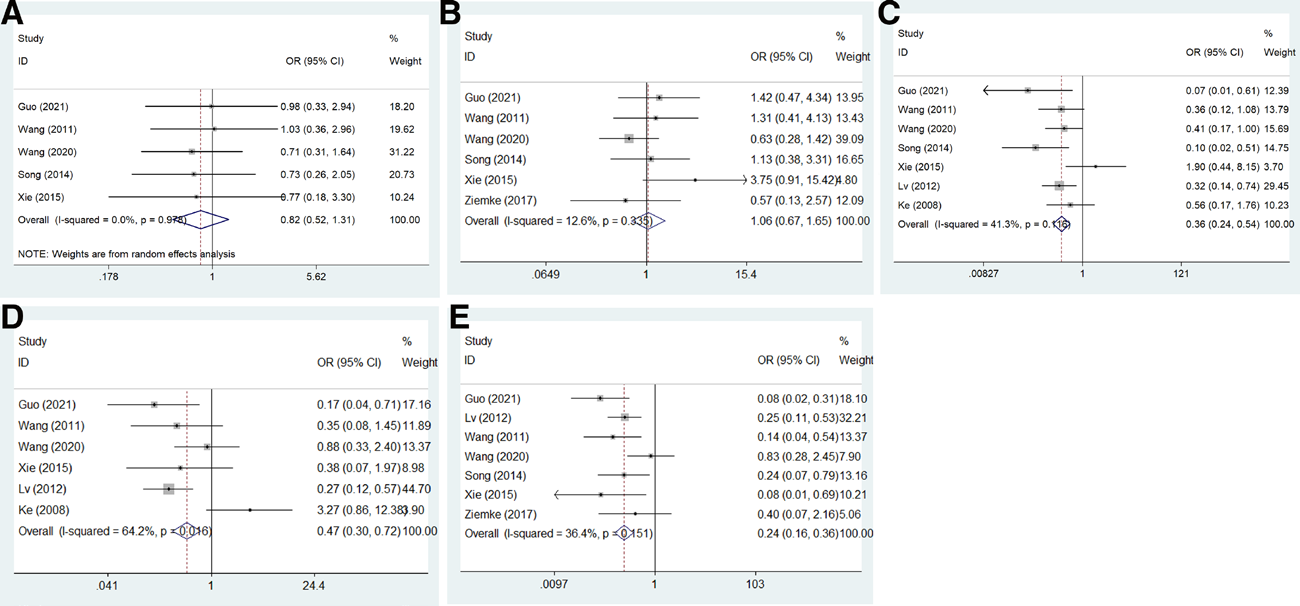
Forest plots showing the correlation between SMAD4 and clinicopathological parameters of NSCLC patients. (A) Age, (B) sex, (C) differentiation, (D) lymph node metastasis, and (E) TNM stage. NSCLC = non-small cell lung cancer, SMAD4 = SMAD family member 4, TNM = tumor node metastasis.

Our meta-analysis was performed only when the correlation of SMAD4 and clinicopathological parameters exceeded 3 studies. The clinicopathological significance of SMAD4 expression in NSCLC patients was detected. Our results showed that SMAD4 expression was associated with tumor differentiation (OR = 0.359, 95% CI: 0.238–0.543, *P* = .000), lymph node metastasis (OR = 0.469, 95% CI: 0.04–0.725, *P* = .001), and tumor node metastasis (TNM) stage (OR = 0.238, 95% CI: 0.156–0.362, *P* = .000) in NSCLC patients. There was no relationship with age (OR = 0.822, 95% CI: 0.515–1.312, *P* = .411) or sex (OR = 1.056, 95% CI: 0.675–1.653, *P* = .811) in NSCLC patients (Table 2; Fig. 2).

**Table 2:** Results of clinical parameters and SMAD4 expression in patients with NSCLC.

Clinical parameters	No. of studies	Overall OR (95% CI)	Heterogeneity test (Q, *I*^2^, *P*)
Age	1, 3, 4, 5, 6	0.822 (0.515–1.312)	0.45, 0.0%, .978 (fixed)
Gender	1, 3, 4, 5, 6, 7	1.056 (0.675–1.653)	5.72, 12.6%, .811 (fixed)
Differentiation	1, 2, 3, 4, 5, 6, 8	2.784 (1.842–4.209)	10.22, 41.3%, .116 (fixed)
N	1, 2, 3, 4, 6, 8	0.469 (0.304–0.725)	13.96, 64.2%, .0016 (random)
TNM	1, 2, 3, 4, 5, 6, 7	0.228 (0.156–0.362)	9.43, 36.4%, .151 (fixed)

NSCLC = non-small cell lung cancer, SMAD4 = SMAD family member 4, TNM = tumor node metastasis.

### 3.3. Publication bias between SMAD4 and clinicopathological parameters

**Figure 3.**
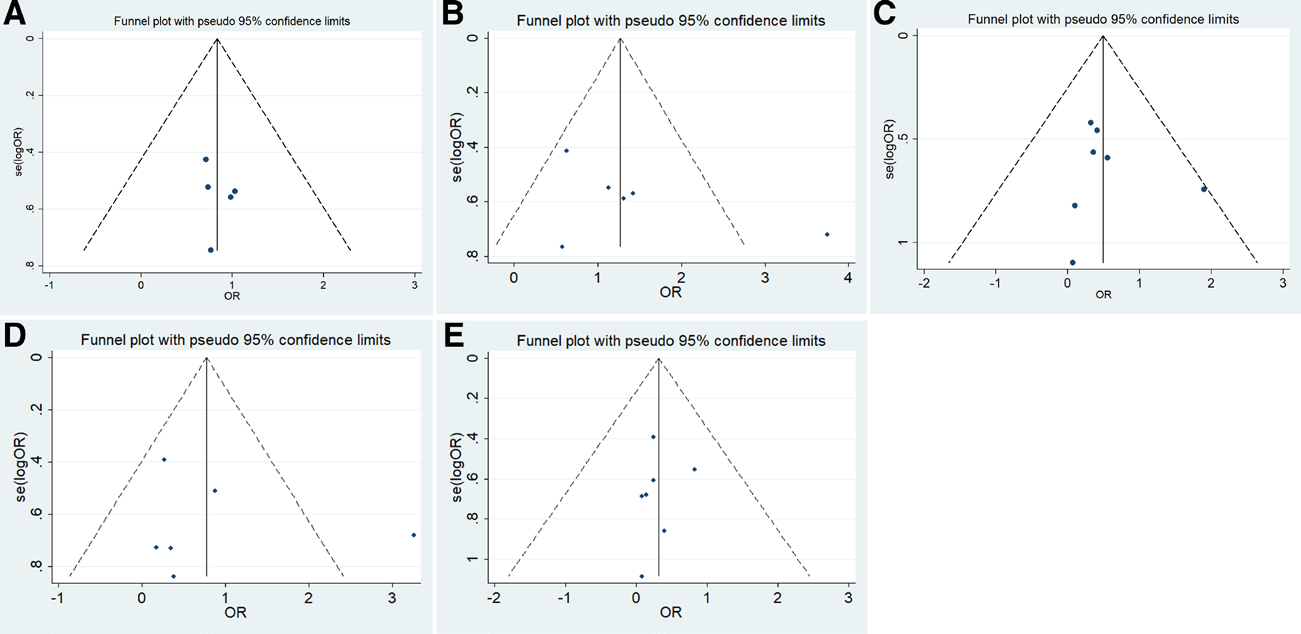
Egger’s funnel plot estimated the publication bias of the correlation between SMAD4 and the clinicopathological parameters of NSCLC patients. (A) Age, (B) sex, (C) differentiation, (D) lymph node metastasis, and (E) TNM stage. NSCLC = non-small cell lung cancer, SMAD4 = SMAD family member 4, TNM = tumor node metastasis.

Next, the publication bias between SMAD4 and clinicopathological parameters was detected by Begg’s and Egger’s tests. Our results showed that Begg’s funnel plots between SMAD4 and clinicopathological parameters (tumor differentiation, lymph node metastasis, TNM stage, age and sex) were symmetric, and the P values of Egger’s test were 0.600, 0.271, 0.587, 0.700, and 0.482, respectively (Fig. 3). Therefore, our results indicated no significant publication bias.

### 3.4. Association between SMAD4 and OS

**Figure 4.**
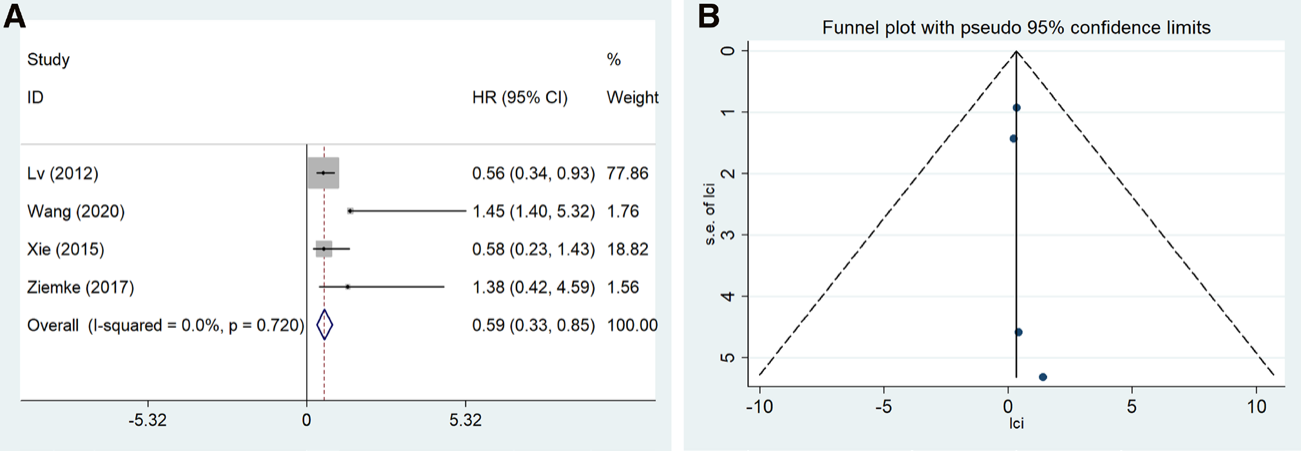
The correlation between SMAD4 and OS of NSCLC patients. (A) forest plots, (B) funnel plot. NSCLC = non-small cell lung cancer, SMAD4 = SMAD family member 4.

Four of our included studies reported the prognostic significance of SMAD4 expression in NSCLC patients. SMAD4 expression was related to good OS (HR = 0.592, 95% CI: 0.332–0.853, *P* = .000), and the results are reported in Figure 4A.

Begg’s funnel plot between SMAD4 and the OS of the NSCLC patients was symmetric, and the *P* value of Egger’s test of OS was 0.350. There was no significant publication bias between SMAD4 and OS for NSCLC patients (Fig. 4B).

### 3.5. Sensitivity analysis

**Figure 5.**
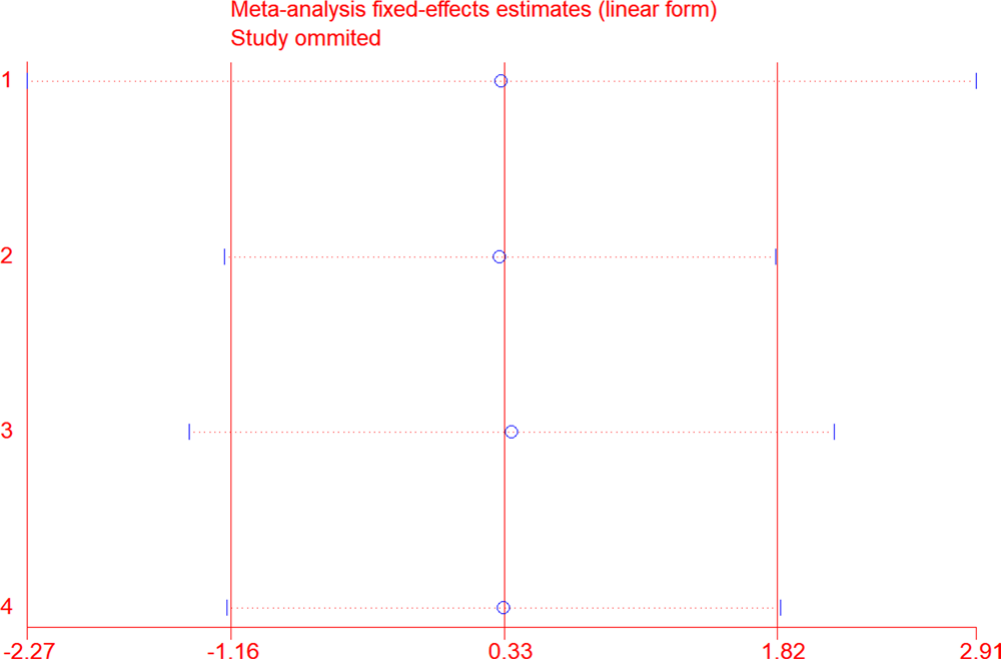
Sensitivity analysis results of SMAD4 and OS of NSCLC patients. NSCLC = non-small cell lung cancer, SMAD4 = SMAD family member 4.

We used sensitivity analysis to evaluate the stability of our results, and the sensitivity analysis indicated that the exclusion of any single included study did not alter the statistical significance of the results. This sensitivity analysis result indicated that the results were stable (Fig. 5).

### 3.6. SMAD4 expression and prognostic potential in NSCLC

**Figure 6.**
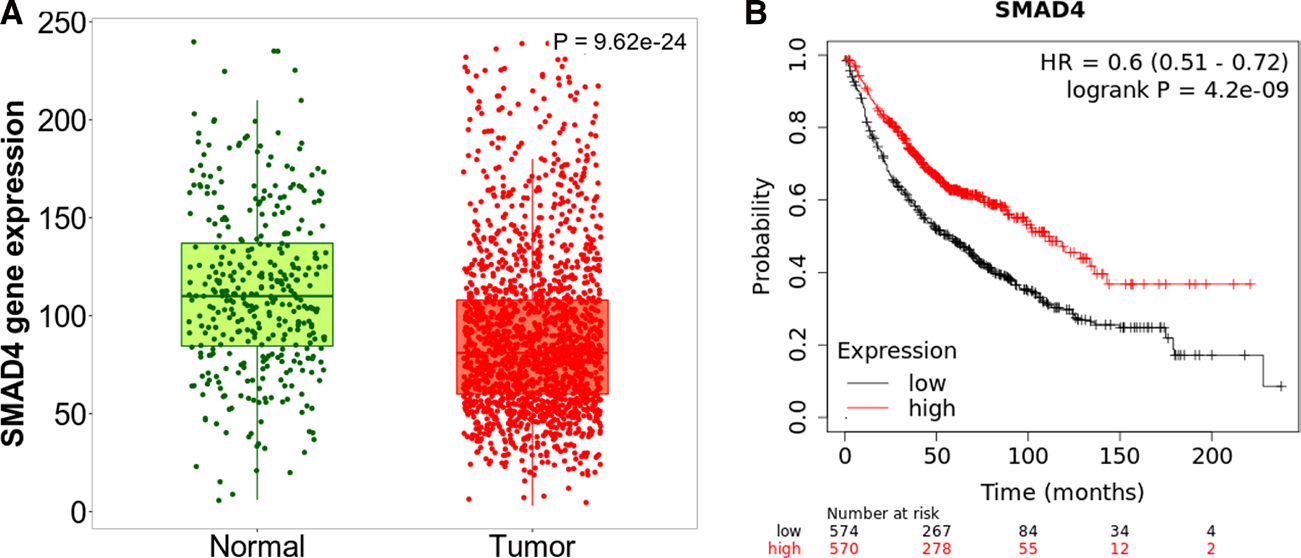
The expression and prognostic potential of SMAD4 in NSCLC in the Kaplan–Meier plotter database. (A) SMAD4 expression was lower in NSCLC tissues than in normal tissues. (B) Kaplan–Meier survival curves comparing high and low SMAD4 expression. NSCLC = non-small cell lung cancer, SMAD4 = SMAD family member 4.

To determine the expression of SMAD4 in NSCLC tissues, the expression of SMAD4 in NSCLC and normal lung tissues was analyzed using the Kaplan–Meier Plotter database. This analysis showed that SMAD4 expression was lower in NSCLC tissue than in normal lung tissue (Fig. 6A).

Next, we investigated whether SMAD4 expression was correlated with the prognosis of NSCLC patients. The results indicated that good prognosis in NSCLC (HR = 0.6, 95% CI = 0.51–0.72, *P* = 4.2 e-9) was correlated with higher SMAD4 expression using the Kaplan–Meier plotter database (Fig. 6B).

## 4. Discussion

NSCLC includes lung squamous cell carcinoma and adenocarcinoma and accounts for more than 80% of lung cancers.^[19]^ With the development of surgery, chemotherapy, molecular targeted therapy and immunotherapy, promising advances in NSCLC treatment have been made, but the prognosis is still poor.^[20,21]^ Recently, the clinicopathological and prognostic significance of SMAD4 expression in NSCLC patients was studied, but the conclusion is still controversial. In our meta-analysis, we found that SMAD4 expression is correlated with tumor differentiation, lymph node metastasis and TNM stage but not with the age or sex of NSCLC patients and SMAD4 expression is lower in NSCLC and is correlated with good OS of NSCLC patients.

Many authors have reported that the levels of SMAD4 expression play important roles in many kinds of cancers.^[22–24]^ The roles of SMAD4 in animal models, where SMAD4 is involved in tumor formation and the development of metastasis in prostate, colorectal and pancreatic cancers, have been confirmed.^[25]^ In lung cancer, SMAD4 activates TGF-β signaling and contributes to tumor metastasis by regulating the TGF-β pathway.^[26]^ Chae et al^[27]^ observed that SMAD4 expression is low in lung cancer tissues and cell lines and that SMAD4 is regulated by miR-27a and involved in the cell proliferation and invasion of lung cancer. Many recent studies have detected a relationship between SMAD4 expression and the clinicopathological parameters of NSCLC. Guo et al^[10]^ investigated the correlation between SMAD4 expression in NSCLC tissues and clinicopathological significance and found that SMAD4 levels were lower in NSCLC tissues, and the low expression of SMAD4 was related to tumor differentiation, lymph node metastasis and the TNM stage of NSCLC patients. However, the relationship between SMAD4 expression and the clinicopathologic parameters of NSCLC patients is still controversial. Ziemke et al^[12]^ retrospectively identified NSCLC patients and assessed SMAD4 expression by immunohistochemistry. They found that SMAD4 expression was low in NSCLC tissues but was not related to the clinicopathologic parameters of NSCLC patients. In this study, we found that SMAD4 is correlated with tumor differentiation, lymph node metastasis and the TNM stage of NSCLC patients but is not correlated with age or sex.

An increasing number of studies have detected a correlation between SMAD4 and survival for many kinds of cancer. As a tumor suppressor gene, SMAD4 is inactivated in approximately 60% of pancreatic adenocarcinomas.^[28]^ The OS of pancreatic adenocarcinoma patients in the SMAD4+ groups was much better than that in the SMAD4− groups.^[29]^ In lung cancer, the recurrence-free survival of patients with higher expression of SMAD4 showed significant improvement.^[30]^ SMAD4 activates TGF-β signaling, and SMAD4 is associated with improved OS in human lung cancer.^[31]^ However, it was unable to demonstrate a relationship between SMAD4 and prognosis in NSCLC patients.^[12]^ Lv et al^[14]^ analyzed SMAD4 expression and prognosis in 150 NSCLC patients and found that Smad4 was correlated with an improved prognosis of NSCLC. However, Xie et al evaluated SMAD4 expression by immunohistochemistry in 85 NSCLC patients and analyzed the relationships between SMAD4 expression and the prognosis of NSCLC patients, finding that there was no significant association between SMAD4 and the OS of NSCLC patients.^[11]^ In this study, we found that SMAD4 was correlated with good OS in NSCLC. Furthermore, in the Kaplan–Meier Plotter database, we detected the expression of SMAD4 in NSCLC and normal lung tissues and analyzed the prognostic significance of SMAD4 expression in NSCLC patients. Interestingly, we reached the same conclusion as in the meta-analysis: SMAD4 expression was lower in NSCLC than in normal lung tissue, and good OS was observed in NSCLC patients with higher SMAD4 expression. However, the conclusion should be confirmed by high-quality and large-sample studies to assess the prognosis of SMAD4 in NSCLC.

There were some limitations in this meta-analysis. First, only studies published in English and Chinese and a small sample of some articles were included in our study. This may have caused heterogeneity and bias and limited the accuracy of our conclusions. High-quality studies and multicenter studies with large sample sizes should be carried out in the future. Second, we used software to estimate HRs and 95% CIs in some of our included studies, which may have biased the conclusions. Moreover, TGFβ has dual functions in normal versus tumors.^[32,33]^ Therefore, the expression of SMAD4 in early disease would have a very different outcome to the disease process than expression in late-stage disease. In our result, our aim of this article is to investigate the relationship between SMAD4 expression and the clinicopathological parameters and prognosis of NSCLC patients, and we found that SMAD4 expression was associated with TNM stage (stages I-II and stages III-IV, OR = 0.238, 95% CI: 0.156–0.362, *P* = .000) in NSCLC patients. It is necessary to further analysis the SMAD4 expression in different I–IV TNM stages. In addition, it has been reported that SMAD4 mutation can be used to identify the NSCLC patients with poor survival, and SMAD4 may be a therapeutic target in NSCLC.^[15]^ These driver mutations would include ras, EGF receptor, p53, etc.^[34]^ In our study, we just analysis the expression of SMAD4 in NSCLC by IHC, and the genes mutations present in the NSCLC tumor samples were not included. Therefore, we may analysis this interesting area in the future, and we added the important limitations in discussion of our revised manuscript.

## 5. Conclusion

In summary, we found that SMAD4 expression is lower in NSCLC and correlated with tumor differentiation, lymph node metastasis, TNM stage and good OS but not with the age or sex of NSCLC patients. SMAD4 may be used as a novel biomarker for predicting the outcomes of NSCLC patients.

## Author contributions

**Conceptualization:** Zhiqiang Li, Yunfei Huang, Rongsheng Zhou, Zhicheng Li, Qitao Yan.

**Data curation:** Zhiqiang Li, Yunfei Huang, Rongsheng Zhou, Zhicheng Li, Qitao Yan.

**Formal analysis:** Zhiqiang Li, Yunfei Huang, Rongsheng Zhou, Zhicheng Li, Qitao Yan.

**Funding acquisition:** Qitao Yan.

**Investigation:** Zhiqiang Li, Yunfei Huang, Rongsheng Zhou, Zhicheng Li, Qitao Yan.

**Methodology:** Zhiqiang Li, Yunfei Huang, Rongsheng Zhou, Zhicheng Li, Qitao Yan.

**Project administration:** Zhiqiang Li, Yunfei Huang, Qitao Yan.

**Resources:** Zhiqiang Li, Yunfei Huang, Rongsheng Zhou, Qitao Yan.

**Software:** Rongsheng Zhou, Zhicheng Li.

**Supervision:** Zhicheng Li, Qitao Yan.

**Validation:** Zhiqiang Li, Yunfei Huang, Rongsheng Zhou, Zhicheng Li, Qitao Yan.

**Visualization:** Zhiqiang Li, Yunfei Huang, Rongsheng Zhou, Qitao Yan.

**Writing – original draft:** Zhiqiang Li, Yunfei Huang, Rongsheng Zhou, Zhicheng Li.

**Writing – review & editing:** Qitao Yan.
